# Asset-Aware and Resilient Trust Management Framework for Industrial IoT Edge Networks

**DOI:** 10.3390/s26123808

**Published:** 2026-06-15

**Authors:** Yufei Wang, Huanhuan Gu, Qian Ye

**Affiliations:** 1 School of Internet of Things Engineering, Wuxi University of Technology, Wuxi 214121, China; 2 School of Cyber Science and Engineering, Nanjing University of Science and Technology, Nanjing 210094, China; guhuanhuan@njust.edu.cn; 3 Nanjing Sinovatio Technology Co., Ltd., Nanjing 211153, China; 4 School of Control Engineering, Wuxi University of Technology, Wuxi 214121, China; yeqian@wxit.edu.cn

**Keywords:** Industrial Internet of Things (IIoT), trust management, edge computing, asset-aware security, fuzzy entropy weight method, whitewashing attacks

## Abstract

Trust evaluation in Industrial Internet of Things (IIoT) edge networks must account for both device behavior and the operational importance of industrial assets. Existing models often apply uniform scoring rules, which may limit their response to semantic attacks and whitewashing behavior while increasing the processing burden on edge devices. This paper presents an Asset-Aware Resilient Trust (ART) framework. ART separates dynamic behavioral credibility from physical asset criticality through a dual-plane architecture. Cross-layer evidence is collected from communication, identity, physical, and semantic interactions. A Fuzzy Triggered-Entropy Weight Method (Fuzzy T-EWM) recalculates evidence weights only when the observed fluctuation exceeds a preset threshold. Trust scores are updated using a Fast-Drop Slow-Rise rule, together with a tolerance margin for routine network jitter. The simulation results show that ART detects stealthy False Data Injection attacks, limits trust recovery after whitewashing behavior, and reduces accumulated computational overhead by 76.4% compared with the Standard EWM baseline. The credibility-weighted aggregation mechanism also limits collusive recommendation manipulation during cold-start evaluation. These results support differentiated trust regulation for IIoT edge networks.

## 1. Introduction

Industry 4.0 relies on the integration of information technology and operational technology, providing the foundation for smart manufacturing environments [1,2]. The Industrial Internet of Things (IIoT) connects sensors, actuators, programmable logic controllers (PLCs), and edge gateways across industrial production systems [3,4]. While this connectivity improves monitoring and automation, it also expands the attack surface. Static perimeter defenses and conventional intrusion-detection systems are often insufficient for identifying internal threats that originate from compromised or previously trusted devices [5].

Trust management has therefore become an important component of Zero Trust security in IIoT edge networks [6]. Instead of relying on a one-time admission decision, Zero Trust continuously evaluates node behavior and adjusts access permissions according to the observed context [7]. This approach can limit unauthorized lateral movement and reduce the influence of compromised devices [8,9]. Recent studies have also emphasized the need for trust mechanisms that can operate efficiently at the edge while supporting timely threat isolation [10,11].

Despite these advances, existing IIoT trust models still face several limitations. First, many approaches focus mainly on communication-level indicators, such as packet loss, delay, or interaction frequency. These indicators are useful for identifying network disruptions, but they may not reveal semantic manipulation. A False Data Injection (FDI) attack can alter physical readings while preserving normal traffic patterns, allowing the malicious behavior to remain undetected by network-centric methods [12,13].

Second, many trust models apply the same update rule to all devices. This uniform treatment does not reflect the operational differences among industrial assets. A deviation produced by a core PLC may have a greater impact on the production process than a similar deviation produced by an auxiliary sensor. Uniform recovery rules also create opportunities for whitewashing and on–off attacks, in which a malicious node gradually restores its trust score after a short period of compliant behavior [14,15,16].

Third, continuous trust recalculation can impose a substantial burden on resource-constrained edge devices. Industrial nodes often operate with limited memory, processing capacity, and energy resources [17]. Recalculating multi-dimensional trust weights at every evaluation slot may therefore reduce the feasibility of long-term deployment.

To address these issues, this paper proposes an ART framework for IIoT edge networks. ART separates dynamic cyber-behavioral credibility from physical asset criticality through a dual-plane architecture. The horizontal plane collects cross-layer evidence from communication, identity, physical, and semantic interactions. The vertical plane introduces the asset value $V_{s}$ as contextual information for trust regulation. The two planes interact through an edge-gateway workflow that combines evidence collection, triggered weighting, direct and indirect trust aggregation, and context-aware decision-making.

The main contributions of this study are summarized as follows:

- Asset-aware trust regulation: ART separates behavioral credibility from physical asset criticality. The asset value $V_{s}$ is incorporated into the trust-update process, allowing devices with different operational roles to be evaluated under differentiated recovery conditions.
- Cross-layer evidence evaluation: The framework combines communication indicators with physical and semantic consistency checks. This design improves the detection of stealthy FDI attacks that may not produce obvious network-level anomalies.
- Resilient asymmetric updating: ART introduces a Fast-Drop Slow-Rise rule with the tolerance margin $\Delta_{tol}$. Significant deviations lead to a rapid trust reduction, while routine network jitter produces only a limited temporary effect. The recovery process is further regulated according to asset value and anomaly severity.
- Lightweight triggered evaluation: The proposed Fuzzy Triggered-Entropy Weight Method (Fuzzy T-EWM) recalculates entropy weights only when evidence fluctuations exceed the asset-aware trigger threshold. During stable periods, cached weights are reused to reduce unnecessary matrix operations.
- Robust recommendation aggregation: A credibility-weighted trimmed mean mechanism is used during indirect trust initialization. This mechanism limits the influence of collusive recommendation manipulation during the cold-start phase.

Simulation results show that ART detects stealthy FDI attacks, limits whitewashing recovery, and reduces accumulated computational overhead by 76.4% compared with the Standard EWM baseline. The evaluation also includes Sybil-style collusive recommendation manipulation and a near-complete black-hole packet-dropping scenario. These results indicate that ART provides an efficient and resilient trust-management approach for heterogeneous IIoT edge networks.

## 2. Related Works

### 2.1. Trust Management in IIoT

Trust management provides an additional security layer for Industrial Internet of Things (IIoT) environments. Early approaches often relied on Bayesian reasoning or fuzzy logic to aggregate service-quality indicators and estimate node reliability [18,19]. These methods are suitable for basic trust evaluation, but their effectiveness may be limited in heterogeneous industrial networks. In particular, many models focus on behavioral observations without explicitly considering the operational roles of physical assets [18].

Industrial devices do not have the same level of criticality. A temporary deviation from an auxiliary sensor and a similar deviation from a core PLC may lead to different operational consequences. Trust regulation should therefore account for both node behavior and asset importance. ART addresses this issue through an asset-aware control plane. The asset value $V_{s}$ is incorporated into the trust-management process, allowing the framework to apply differentiated trigger thresholds and recovery conditions to devices with different operational roles.

### 2.2. Defense Against Malicious Behaviors

Trust-management mechanisms in IIoT networks must respond to attacks that may not be visible from communication indicators alone. Two types of malicious behavior are particularly relevant to this study.

- Semantic Threats: Many security mechanisms rely mainly on communication-level indicators, such as packet delivery, delay, or interaction frequency. These indicators are useful for detecting network disruptions, but they may not reveal whether the transmitted physical data remain consistent with the actual operating process. In a False Data Injection (FDI) attack, an adversary may alter sensor readings while preserving apparently normal traffic patterns [20,21,22]. Detecting this behavior requires physical and semantic consistency checks in addition to conventional network monitoring [21].
- Whitewashing and On–Off Attacks: Symmetric trust-update models may remain vulnerable to whitewashing and on–off attacks [23,24,25]. In an on–off attack, a malicious node alternates between abnormal and compliant behavior. If trust decreases and recovers at similar rates, a short period of normal activity may allow the node to restore its score too quickly and re-enter the network.

ART addresses these issues through cross-layer evidence evaluation and an asymmetric Fast-Drop Slow-Rise rule. Communication, identity, physical, and semantic indicators are jointly considered during trust evaluation. Significant deviations activate the Fast-Drop branch, while the tolerance margin $\Delta_{tol}$ limits unnecessary penalties caused by routine network jitter. Trust recovery is regulated by the asset value $V_{s}$ and the anomaly-severity factor κ. This design limits rapid trust restoration after potentially malicious behavior while preserving recovery from short-term non-malicious disturbances.

### 2.3. Computational Efficiency at the Edge

Trust fusion at the edge must remain lightweight because IIoT nodes usually have limited computational power, memory, and energy resources [17,26]. Some fuzzy trust and security models use predefined evaluation rules or linguistic variables [18,19]. More generally, fuzzy decision methods may involve expert judgments and the selection of fuzzy scales or membership functions, which can affect the resulting weights [27]. Entropy Weight Methods (EWM) provide data-driven weight allocation based on the information carried by the evaluation indicators [28]. In the baseline implementation used in this study, the entropy weights are recalculated at each evaluation slot. This repeated calculation increases the processing burden when the network remains stable. Event-triggered mechanisms have also been applied to resource-aware secure control under FDI attacks [29]. In ART, the entropy weights are recalculated only when the evidence fluctuation exceeds the trigger threshold. During stable periods, the previously calculated weights are reused to avoid unnecessary matrix operations. The simulation results show that this strategy reduces the accumulated computational overhead by 76.4% compared with the Standard EWM baseline while maintaining timely anomaly detection.

### 2.4. Comparison Summary

We directly compare our ART framework with the rule-based fuzzy models [18,19], a Standard EWM baseline [28], and ML-based models [26,30,31,32]. The comparison considers weight allocation, update mode, edge-processing burden, asset awareness, trust-update logic, detection scope, and noise tolerance. These aspects are relevant to IIoT edge deployment, where trust evaluation must remain lightweight while accounting for device criticality and abnormal behavior.

As summarized in Table 1, the listed approaches have different characteristics. Rule-based fuzzy models are relatively simple to implement, but their performance may depend on predefined rules and parameter settings. Standard EWM provides data-driven entropy weighting [28], whereas ML-based models can capture complex behavioral patterns but may incur additional inference costs at the edge [26,30,31,32].

ART combines entropy-based weight allocation with event-triggered updates and cached weights. It also incorporates a CIA-based asset value $V_{s}$, cross-layer H1–H3 evidence fusion, and a context-aware asymmetric update rule. The adaptive threshold and tolerance margin $\Delta_{tol}$ are used to limit the influence of minor environmental fluctuations.

The same trend can also be observed in recent studies on cyber-physical resilience. Researchers are no longer concerned only with detecting anomalies after damage has occurred. More attention is being paid to how industrial systems can continue operating under sophisticated deception attacks [33]. Some researchers attempt to solve this problem through proactive defense mechanisms. Moving Target Defense (MTD) is a representative example [34]. By continuously changing system parameters, it makes it more difficult for attackers to obtain an accurate understanding of the target system. However, no proactive mechanism can guarantee that every attack will be blocked in advance. Once an attacker successfully bypasses the first layer of protection, the system still needs a reliable way to judge whether a node can be trusted. ART focuses on this stage. Through semantic consistency verification and asset-aware trust control, suspicious nodes can be identified and restricted before their influence spreads further in the industrial network.

## 3. The Proposed ART Architecture

Most conventional trust-management models evaluate nodes using a uniform scoring process. Such an approach does not fully reflect the heterogeneous roles of devices in IIoT environments. A temporary deviation generated by an auxiliary sensor and a similar deviation generated by a core PLC may have different operational consequences. To address this issue, ART adopts a dual-plane architecture that separates dynamic cyber-behavioral credibility from physical asset criticality.

**Figure 1:**
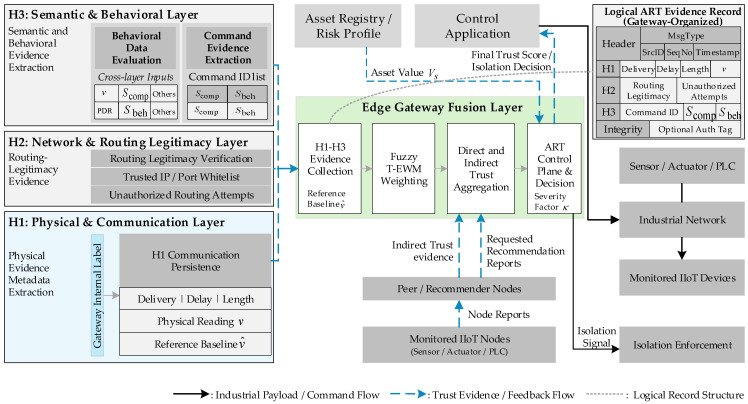
Architecture of the ART framework and the logical evidence-exchange flow. Solid arrows indicate industrial payload and command transmission, dashed arrows indicate trust-evidence reporting and feedback propagation, and dotted lines indicate the logical record structure. PDR denotes the Packet Delivery Ratio.

As illustrated in Figure 1, the horizontal plane collects and evaluates cross-layer evidence, while the vertical plane provides the asset value $V_{s}$ as contextual information for trust regulation. The two planes interact at the edge gateway. The gateway performs evidence collection, Fuzzy T-EWM weighting, direct and indirect trust aggregation, and context-aware decision-making. The resulting trust score and isolation decision are forwarded to the control application and the isolation-enforcement module.

ART does not replace the application-level packet format of the deployed IIoT protocol. Industrial payloads and control commands continue to follow the underlying communication protocol. Trust evaluation is performed at the edge by extracting communication metadata, identity information, and semantic indicators from observed interactions. These observations are organized as a logical ART evidence record at the gateway. The record contains a common header, H1–H3 evidence fields, and an optional integrity field. The common header includes the message type, source identifier, sequence number, and timestamp. The integrity field is reserved for an authentication tag when cryptographic verification is enabled.

### 3.1. Orthogonal Two-Plane Overview

ART operates across two intersecting dimensions:

- Horizontal Plane (Dynamic Behavior Evaluation): This plane captures real-time interaction evidence across the communication, identity, physical, and semantic dimensions. The evidence is organized into the H1–H3 layers and processed by the edge gateway. The objective is to characterize the current behavior of a node rather than relying on a single communication indicator.
- Vertical Plane (Asset-Aware Control): This plane introduces the normalized asset value $V_{s}$ as contextual information. The value reflects the operational criticality of a device within the industrial process. In the current simulations, the asset values remain fixed to maintain controlled experimental conditions. In practical deployments, they can be periodically recalibrated according to device roles, asset inventories, process dependencies, and operational risk assessments.

The two planes are orthogonal but interdependent. The horizontal plane determines how a node behaves, while the vertical plane determines how strictly the observed behavior should be regulated. The ART control plane combines these two dimensions during trust updating and isolation decision-making.

### 3.2. Dynamic Behavior Credibility Evaluation Plane

The horizontal plane is divided into three evidence layers.

- H1-Physical and Communication Layer: The H1 layer collects packet-delivery status, transmission delay, packet length, and the physical reading v. These indicators characterize communication persistence and physical-data availability. The reference baseline $\hat{v}$ is maintained or retrieved at the edge gateway rather than transmitted as an application-payload field. It is used to evaluate whether the observed physical reading remains consistent with the recent operating state.
- H2-Network and Routing Legitimacy Layer: The H2 layer evaluates whether a node forwards data through authorized communication paths. The observed destination is checked against a locally maintained whitelist of trusted Internet Protocol (IP) addresses and ports. Unauthorized routing attempts reduce the routing-legitimacy score and may indicate abnormal data transmission or routing manipulation.
- H3-Semantic and Behavioral Layer: The H3 layer evaluates command evidence and behavioral conformity. The command-compliance score $S_{comp}$ reflects whether the observed operation follows the expected industrial logic, while the behavioral-conformity score $S_{beh}$ captures deviations from the normal operating pattern. Selected H1 and H2 indicators, such as the physical reading and packet-delivery ratio, are also used as cross-layer inputs for behavioral evaluation.

The extracted indicators are organized as a logical ART evidence record. The H1 fields contain the packet-delivery result, delay, packet length, and physical reading v. The H2 fields contain the routing-legitimacy score and the number of unauthorized routing attempts. The H3 fields contain the command identifier, $S_{comp}$, and $S_{beh}$. This gateway-organized record is used for trust evaluation and does not modify the original industrial payload.

### 3.3. Global Asset Value Control Plane

The vertical plane provides the asset value $V_{s}$, which represents the operational criticality of node s. The normalized value is defined as $V_{s}\in [0,1]$. In the simulations, PLC nodes are assigned $V_{s}=0.95$ to represent highly critical assets, while lower values are used for less critical devices. This setting is adopted for scenario differentiation and does not restrict the theoretical range of the model.

The asset value is maintained by the asset registry or risk profile and is provided to the ART control plane as contextual information. It is not transmitted as part of each logical ART evidence record. The asset value affects the trust-update process by regulating the recovery behavior of a node after a deviation. Nodes with higher asset values are evaluated under stricter recovery conditions because abnormal behavior from these devices may have a greater influence on the industrial process.

The severity factor κ is handled separately. It is determined during the trust-update process according to the observed anomaly and is not treated as a static asset attribute. In this way, ART distinguishes the operational importance of a device from the severity of its current behavior.

### 3.4. Asset-Aware Workflow

The two planes interact through an edge-oriented workflow.

- Evidence Collection: The edge gateway collects H1–H3 evidence from observed interactions. The gateway also maintains the reference baseline $\hat{v}$ required for physical-consistency evaluation.
- Triggered Weighting: Fuzzy T-EWM monitors the evidence fluctuation ΔE. When the trigger condition is not satisfied, the cached weights are reused. When the fluctuation exceeds the asset-aware threshold, the entropy weights are recalculated. This mechanism avoids repeated matrix operations during stable operating periods.
- Trust Aggregation: The gateway combines direct evidence with indirect trust information. Recommendation reports are requested from peer nodes when indirect trust evaluation is required. The credibility-weighted aggregation mechanism limits the influence of manipulated recommendations during the cold-start phase.
- Context-Aware Decision: After aggregation, the ART control plane applies the asymmetric trust-update rule. The update process considers the asset value $V_{s}$, the severity factor κ, and the tolerance margin $\Delta_{tol}$. The final trust score and isolation decision are forwarded to the control application. When isolation is required, the corresponding signal is sent to the isolation-enforcement module.

In Figure 1, solid arrows indicate industrial payload and command transmission, dashed arrows indicate trust-evidence reporting and feedback propagation, and dotted lines indicate the logical record structure.

## 4. Methodology and Mathematical Modeling

### 4.1. Quantification and Calculation of Dynamic Behavior Credibility Metrics

Following the horizontal plane set out in Section 3, we now turn those abstract node behaviors into computable trust evidence. We divide this quantitative process into three specific layers: physical and communication evidence, network and routing-legitimacy evidence, and semantic and behavioral evidence.

#### 4.1.1. Physical and Communication Layer Evidence (H1)

Interaction Success Rate (η): We use a standard Bayesian Beta distribution model to calculate the basic reliability of node cooperation. Its definition is as follows:

(1) $$\eta =\frac{\alpha +1}{\alpha +\beta +2}$$

where α and β represent the total number of successful and failed historical interactions within the current time window, respectively.

Transmission Delay ($T_{delay}$): Network delay may result from temporary congestion or persistent abnormal behavior. To distinguish these cases, the delay trust $T_{delay}$ is calculated using the following piecewise exponential decay function:

(2) $$T_{delay}=\{\begin{array}{c} 1,\;\;\;\;\;\;\;\;\;\;\;\;\;\;\;\;\;\;\;\;\;\;\;\;\;\;\;\;\;\;\;\;\;\;\;\;\;\;\;\;\;\;\;\;\;\;{Delay}_{ij}<\;\;u\;\;\\ \left(1-\varphi r\right){C_{1}}^{\frac{{Delay}_{ij}-u}{u}},\;\;u\leq \;\;{Delay}_{ij}<\xi u\\ \left(1-\varphi r\right)C_{1}^{\xi -1}{C_{2}}^{\frac{{Delay}_{ij}-\xi u}{u}},\;\;\;\;\;\;\;\;\;\;\;\;\;\;\;\;{Delay}_{ij}\geq \xi u \end{array}\;\;\;$$

where ${Delay}_{ij}$ is the current transmission delay between node i and j, u is the upper bound of the normal delay range. The parameter ξ>1 defines the boundary for severe delays. In this study, ξ=2 is used as a practical default to distinguish temporary congestion from prolonged timeouts. Delays within [u,ξu) are treated as moderate deviations, whereas delays exceeding ξu are classified as severe deviations. The value of ξ can be recalibrated according to the delay distribution and timeout tolerance of the deployed industrial network.

The parameters $C_{1}$ and $C_{2}$ are configurable decay bases for moderate and severe delays, respectively, with 0 < $C_{2}$ < $C_{1}$ < 1. A practical default setting is $C_{1}=0.8$ and $C_{2}=0.4$. The larger value of $C_{1}$ produces a gradual decline during temporary congestion, whereas the smaller value of $C_{2}$ results in a steeper decline during prolonged timeouts. These values can also be recalibrated for the target network. The historical timeout ratio r and the trend weight φ are bounded within [0,1].

Energy Adaptation Metric (ε): Sudden increases in energy consumption may indicate abnormal workloads, such as illicit cryptomining. This metric evaluates whether the node can sustain its expected task-execution pattern. We compute the comprehensive energy trust ε by fusing the raw energy reserve ($\varepsilon_{re}$) with an adaptation factor ($\varepsilon_{adapt}$):

(3) $$\varepsilon =\rho_{re}\cdot \varepsilon_{re}+\rho_{adapt}\cdot \varepsilon_{adapt}$$

where $\rho_{re}$ and $\rho_{adapt}$ are weight factors. Each individual part is quantified as follows:

(4) $$\varepsilon_{re}=min\left(\frac{{RE}_{j}}{{PE}_{j}}\times exp\left(-k_{1}\times \frac{{CE}_{j}}{{PE}_{j}}\right),1\right)$$

(5) $$\varepsilon_{adapt}=exp\left(-k_{2}\cdot \frac{\left|{CE}_{j}-{CE}_{theo}\right|}{{CE}_{theo}}\right)$$

where ${RE}_{j}$ and ${PE}_{j}$ are the residual and rated energy of node j. The variable ${CE}_{j}$ denotes the current energy consumption rate, ${CE}_{theo}$ represents the theoretical workload consumption, and $k_{1}$, $k_{2}$ act as sensitivity adjustment coefficients.

Physical Payload Consistency and Logic Residual Detection: Communication metrics alone are not sufficient for identifying every abnormal interaction. In an FDI attack, the packet-delivery rate and packet length may remain normal even though the sensor reading has been altered. ART therefore checks the physical value carried by the packet against its reference baseline. Packet length is retained as a structural indicator, while the logic residual reflects the deviation of the observed reading from the expected operating state. The payload-consistency score is calculated as follows:

(6) $$p\left(t\right)=exp\left(-\left[\omega_{l}({\frac{l-l_{th}}{l_{th}})}^{2}+\omega_{v}{\left(\frac{v-\hat{v}}{\sigma_{v}}\right)}^{2}\right]\right)$$

where l and $l_{th}$ denote the actual packet length and the expected packet length, respectively. The variable v represents the real-time physical reading, while $\hat{v}$ denotes the corresponding reference baseline under normal operating conditions. The parameter $\sigma_{v}$ is the standard deviation of the physical variable estimated from stable-state observations. In Equation (6), the deviation between v and $\hat{v}$ is normalized by $\sigma_{v}$. Fluctuations comparable to the normal noise level therefore produce limited residual values, whereas persistent or abnormal deviations result in a stronger reduction in the payload-consistency score.

A practical challenge is distinguishing malicious False Data Injection (FDI) attacks from normal industrial fluctuations. In real production environments, not every deviation indicates an attack. Sensor aging, environmental disturbances, and maintenance operations can all affect physical measurements. Therefore, the trust evaluation process should not react to every abnormal reading immediately.

First, sensor drift usually develops gradually. Although the baseline $\hat{v}$ changes over time, the instantaneous residual $v-\hat{v}$ normally remains within the statistical confidence range defined by $3\sigma_{v}$. These deviations are evaluated against the cached reference state in Equation (17) and are further absorbed by the environmental tolerance margin $\Delta_{tol}$ during trust updating Equation (27). As a result, normal industrial jitter is less likely to trigger unnecessary trust penalties.

FDI attacks exhibit a different pattern. In order to manipulate the physical process while remaining hidden at the network layer, attackers often inject abrupt value changes or maintain abnormal deviations for an extended period. Such behavior is difficult to explain through normal process dynamics. Once the residual continuously exceeds the $3\sigma_{v}$ boundary, the payload consistency score p(t) decreases rapidly, causing the trust evaluation mechanism to activate the Fast-Drop penalty. To balance communication-level abnormalities and physical-semantic inconsistencies, the final payload assessment combines the sensitivity weights $\omega_{l}$ and $\omega_{v}$, where (${\omega_{l}+\omega }_{v}=1$).

#### 4.1.2. Network and Routing Legitimacy Layer Evidence (H2)

The H2 layer evaluates whether the observed data-forwarding behavior remains within the authorized network boundary. Industrial edge nodes frequently transmit processed data to gateways or neighboring devices. An unexpected destination may indicate routing manipulation or unauthorized data transmission.

Routing Legitimacy Verification: ART compares the observed destination with a locally maintained whitelist of trusted IP addresses and ports. Routing legitimacy is calculated as follows:

(7) $$R_{legit}=max\left(0,\;1-\;\gamma_{route}\cdot N_{viol}\right)$$

where $N_{viol}$ denotes the number of unauthorized routing attempts, such as attempts to transmit data to an unverified external address. The parameter $\gamma_{route}$ is the penalty coefficient applied to each violation. A smaller legitimacy score indicates a greater deviation from the authorized routing policy.

#### 4.1.3. Semantic and Behavioral Layer Evidence (H3)

The H3 layer focuses on the meaning of the observed operation. A node may appear normal at the communication layer while issuing an unauthorized command or deviating from its expected task pattern. ART evaluates these cases through command compliance and behavioral conformity.

Command Compliance Evaluation: We enforce functional limits using a rigid Privilege Matrix PM. Suppose U represents the edge nodes and CMD be the set of all possible industrial control commands (e.g., Read, Write, Reset). The compliance status of a command c issued by node s is defined by a mapping function f: U×CMD→{0,1}. Unauthorized control commands are particularly dangerous in industrial systems because they can directly affect the physical process. Therefore, command compliance must be considered during trust evaluation. We define the compliance score $S_{comp}\;$ as follows:

(8) $$S_{comp}=\{\begin{array}{c} 1,\;\;\;\;\;\;\;\;\;\;\;\;\;\;\;\;\;\;\;\;\;\;\;\;\;\;\;\;\;\;\;\;\;\;\;\;\;\;\;\;\;\;\;\;\;\;\;\;\;\;\;\;\;\;if\;c\;\in {PM}_{s}\\ \max\left(0,1-\delta \cdot \exp\left(V_{s}\right)\right),if\;c\;\notin \;{PM}_{s}\; \end{array}$$

where ${PM}_{s}$ contains all authorized commands for node s, and δ is the baseline penalty factor. The lower-bound constraint max(0, ·) is added to ensure that the score always remains within the valid trust range. This prevents abnormal values from appearing when the penalty becomes large. In many industrial scenarios, not all unauthorized commands create the same level of risk. Sending an illegal command to a temperature sensor is very different from sending the same command to a PLC that controls a critical production process. For this reason, the penalty is directly linked to the asset value $V_{s}$. As the importance of the target asset increases, the trust score drops more rapidly. This allows the framework to react more aggressively when high-value assets are involved.

Behavioral Conformity and Anomaly Detection: Attackers often use “low-and-slow” probing to map a network without tripping traditional alarms. To catch these stealthy reconnaissance or brute-force attempts, we measure behavioral deviations against a learned historical baseline:

(9) $$S_{beh}=max\left(0,1-\sigma \cdot \frac{\left|{Freq}_{curr}-{Freq}_{avg}\right|}{{Freq}_{avg}}\right)$$

where ${Freq}_{curr}$ represents the current command frequency and ${Freq}_{avg}$ is the long-term average. The sensitivity coefficient σ can be configured according to the operating pattern of the monitored device. A larger value may be used for deterministic control nodes with regular communication cycles, such as robotic arms. For event-triggered sensors, a smaller value allows the model to tolerate short-term traffic bursts associated with normal alarm conditions.

### 4.2. Trust Aggregation via Fuzzy T-EWM

A single indicator is not sufficient to characterize the trustworthiness of an edge node. ART therefore combines multiple evidence types through the Fuzzy Triggered-Entropy Weight Method (Fuzzy T-EWM).

#### 4.2.1. Construction of Fuzzy Evaluation Matrix

We define n types of trust evidence $E=\left\{E_{1},E_{2},{\ldots ,E}_{n}\right\}$ (e.g., success rate, delay, residual energy) and m specific evaluation grades $G=\left\{G_{1},{\ldots ,G}_{m}\right\}$ (e.g., $G_{1}$: Very Trustworthy, $G_{3}$: Untrustworthy). Then we build a fuzzy evaluation matrix $R={\left[r_{ij}\right]}_{n\times m}$, in which the element $r_{ij}\in [0,1]$ represents the membership degree of the i-th evidence with respect to the j-th grade. To bridge the gap between raw quantitative metrics and fuzzy qualitative evaluations, we map the raw evidence $E_{i}$ through standard membership functions (such as Gaussian or trapezoidal distributions).

The trust evidence collected from the three horizontal planes (H1, H2, and H3) does not share the same physical meaning or numerical range. For example, communication delay is measured in milliseconds, residual energy is represented as a percentage, and semantic residuals reflect deviations from physical process expectations. If these indicators are used directly, some evidence may dominate the evaluation simply because of its scale rather than its actual importance. Therefore, all raw evidence is first converted into a common fuzzy evaluation space. We define a three-level evaluation set $V\;=\;\{v_{1}\;(Trustworthy),\;v_{2}\;(Uncertain),\;v_{3}\;(Untrustworthy)\}$. Different mapping functions are used according to the characteristics of the evidence.

(1) Continuous Metrics (e.g., communication delay, energy consumption, and semantic residuals).

For these indicators, smaller values generally indicate a more stable operating condition. We use trapezoidal and triangular membership functions to describe the transition between different trust levels. Let x denote the observed evidence value. The parameter $\alpha_{mem}$ represents the upper boundary of normal operation, while $\beta_{mem}$ denotes the anomaly threshold beyond which the evidence begins to lose credibility. The membership functions for the three grades are defined as follows:

(10) $$v_{1}\left(x\right)=\{\begin{array}{lll} 1 & , & x\leq \alpha_{mem}\\ \frac{\beta_{mem}-x}{\beta_{mem}-\alpha_{mem}} & , & \alpha_{mem}<x<\beta_{mem}\\ 0 & , & x\geq \beta_{mem} \end{array}$$

(11) $$v_{2}\left(x\right)=\{\begin{array}{lll} 0 & , & x\leq \alpha_{mem}\;or\;x\geq \gamma \\ \frac{x-\alpha_{mem}}{\beta_{mem}-\alpha_{mem}} & , & \alpha_{mem}<x\leq \beta_{mem}\\ \frac{\gamma -x}{\gamma -\beta_{mem}} & , & \beta_{mem}<x<\gamma \end{array}$$

where γ denotes the tolerance boundary and is usually selected as $\gamma >\beta_{mem}$.

(12) $$v_{3}\left(x\right)=\{\begin{array}{lll} 0 & , & x\leq \beta_{mem}\\ \frac{x-\beta_{mem}}{\gamma -\beta_{mem}} & , & \beta_{mem}<x<\gamma \\ 1 & , & x\geq \gamma \end{array}$$

The parameters ($\alpha_{mem},\beta_{mem},\;\gamma$) are obtained from historical observations collected in the IIoT edge environment. In this way, the membership functions reflect the actual operating characteristics of the monitored process rather than relying on manually selected thresholds.

(2) Discrete/Logical Metrics (e.g., routing legitimacy).

Some indicators do not vary continuously. A typical example is routing legitimacy in the H2 plane, where the routing path either follows the authorized topology or it does not. For this type of evidence, a discrete membership function is sufficient. When the routing behavior is legitimate (x=0), the evidence is treated as fully trustworthy $v_{1}\left(x\right)=1,v_{2}\left(x\right)=0,\;v_{3}\left(x\right)=0$. When an unauthorized route modification is detected (x = 1), the evidence is directly classified as untrustworthy $v_{1}\left(x\right)=0,v_{2}\left(x\right)=0,\;v_{3}\left(x\right)=1$.

After this mapping process, all evidence can be represented within the same fuzzy evaluation framework. The resulting fuzzy evaluation matrix $R={\left[r_{ij}\right]}_{n\times m}$ provides a consistent basis for the subsequent entropy-weight calculation, regardless of the original units or physical meanings of the indicators.

Finally, the matrix R is normalized into a probability matrix $P={\left[p_{ij}\right]}_{n\times m}$:

(13) $$p_{ij}=\frac{r_{ij}}{\sum_{j=1}^{m}r_{ij}}$$

Here $p_{ij}$ denotes the normalized probability distribution of the i-th evidence across the m grades.

#### 4.2.2. Entropy Calculation and Weight Allocation

Information entropy is used to quantify the dispersion of each evidence type across the evaluation grades. If an evidence metric is distributed across several grades, its entropy is higher and its discriminative contribution is lower. It is therefore assigned a smaller weight. The formula for calculating the information entropy $H_{i}\in [0,1]$ as follows:

(14) $$H_{i}=-\frac{1}{\ln\left(m\right)}\sum_{j=1}^{m}p_{ij}\ln\left(p_{ij}\right)$$

(Note: If $p_{ij}$ = 0, we mathematically define $p_{ij}\ln\left(p_{ij}\right)=0$).

After obtaining this entropy, we calculate the objective weight $w_{i}$ through the variation coefficient ${d_{i}=1-H}_{i}$:

(15) $$w_{i}=\frac{d_{i}}{\sum_{k=1}^{n}d_{k}}=\frac{1-H_{i}}{n-\sum_{k=1}^{n}H_{k}}$$

This formula ensures that all weights add up to 1 ($\sum_{i=1}^{n}w_{i}=1$). Therefore, Evidence metrics with lower entropy receive larger weights in the aggregated trust score. Then we combine these objective weights to get the instantaneous aggregate trust score T(t):

(16) $$T\left(t\right)=\sum_{i=1}^{n}w_{i}\cdot E_{i}$$

Here $E_{i}$ represents the quantified credibility score of the corresponding dimension.

#### 4.2.3. Triggered Calculation Mechanism

Recalculating the entropy-weight matrix at every evaluation slot increases the processing burden at the edge. ART therefore reuses cached weights during stable periods and performs a full recalculation only when the trigger condition is satisfied.

- Steady-State Cache: During stable operating periods, the framework reuses the weights obtained from the most recent triggered recalculation. Retrieving the cached weights requires O(1) time, while the complete stable-slot evaluation remains O(n) because the current evidence vector must still be compared with the cached reference state.
- Anomaly Trigger: To avoid unnecessary weight updates, ART compares the current evidence vector E(t) with the cached reference state $E^{ref}\left(t-1\right)$, which stores the evidence vector from the most recent triggered recalculation. The evidence fluctuation coefficient is defined as follows:
  (17) $$∆E={\left\|E\left(t\right)-E^{ref}\left(t-1\right)\right\|}_{\infty }=\underset{1\leq i\leq n}{\max}\left|E_{i}\left(t\right)-E_{i}^{ref}\left(t-1\right)\right|$$
  where n is the number of evidence types. A larger value of ∆E indicates a greater deviation from the cached operating state. This calculation does not require a historical observation window.
  To support the asset-aware design of ART, the trigger threshold is adjusted according to the asset value of the node. The recalculation threshold for node s is defined as follows:
  (18) $$\Gamma \left(s\right)=\Gamma_{base}\left(1-\mu \cdot V_{s}\right)$$
  where $\Gamma_{base}$ is the baseline tolerance threshold and μ is the sensitivity coefficient. In our simulations, $\Gamma_{base}$ is set to 0.15 and μ is set to 0.5. The baseline value $\Gamma_{base}=0.15$ is selected from the sensitivity analysis in Section 5.2.6 as a balanced setting between recalculation frequency, detection responsiveness, and false alarm control.
  Nodes with higher asset values operate under stricter thresholds, while auxiliary nodes are allowed greater tolerance to routine fluctuations. If ∆E>Γ(s), the entropy weights are recalculated and both the cached weights and the reference evidence state are refreshed. Otherwise, the framework retains the cached values for the next evaluation slot.

Algorithm 1 is the pseudocode of the Fuzzy Triggered-Entropy Weight Method (Fuzzy T-EWM).
**Algorithm 1.** Fuzzy Triggered-Entropy Weight Method (Fuzzy T-EWM)**Input**: Evidence set $E=\left\{E_{1},E_{2},{\ldots ,E}_{n}\right\}$, Fuzzy matrix $R_{n\times m}$, Threshold Γ, Cached weights $W_{cache}$, Cached reference evidence state $E^{ref}$. **Output**: Instantaneous aggregated trust score T(t), Updated cached weights $W_{cache}$; Updated reference evidence state $E^{ref}$.
1.    **Calculate** evidence fluctuation coefficient ΔE using Equation (17)
2.    **If** ΔE>Γ(s) **then**    // Trigger weight recalculation
3.     **Normalize** matrix R to probability matrix P using Equation (13);
4.     **Calculate** information entropy $H_{i}$ for each evidence using Equation (14);
5.     **Compute** objective weights $\;w_{i}$ via variation coefficient ${d_{i}=1-H}_{i}$ using Equation (15);
6.     **Update** $W_{cache}\leftarrow \left\{w_{1},\cdots ,w_{n}\right\}$;
7.     **Update** $E^{ref}\leftarrow E\left(t\right)$;
8.    **End if**
9.    **Retrieve** $W_{current}\leftarrow W_{cache}$; // Cached-weight retrieval requires O(1) time
10.  **Compute** aggregated score $T\left(t\right)=\sum_{i=1}^{n}w_{i}\cdot E_{i}$ using Equation (16);
11.  **Return** T(t), $W_{cache}$, and $E^{ref}$

At the first evaluation slot, the entropy weights are calculated directly, and the initial evidence vector is stored as $E^{ref}$. In subsequent slots, the cached reference state is refreshed only when the trigger condition is met.

Theoretical Complexity Analysis: Let d denote the number of evidence types and q denote the number of observations used to construct the fuzzy evaluation matrix $R\in R^{q\times d}$. At each evaluation slot, ART compares the current evidence vector with the cached reference state. Calculating the evidence fluctuation ΔE(t) requires O(d) time. Retrieving the cached weights requires O(1) time, and calculating the aggregated trust score requires O(d) time. Therefore, the complete stable-slot evaluation has a time complexity of O(d).

When ΔE(t)>Γ(s), the entropy weights are recalculated. Matrix normalization and entropy calculation require O(qd) time, while weight generation and cache refresh require O(d) time. The total complexity of a triggered slot is therefore O(qd+d), which simplifies to O(qd).

Let p∈[0,1] denote the probability that the trigger condition is satisfied in an evaluation slot. The average time complexity per slot is O(d+pqd).

The recursive Fast-Drop and Slow-Rise trust updates require only a fixed number of scalar operations and therefore have a complexity of O(1) for each node. The additional cache stores the reference evidence state and the most recently calculated weights, requiring O(d) memory. The fuzzy evaluation matrix requires O(qd) storage.

For the fixed evidence dimensions used in the simulations, the stable-slot processing cost remains bounded and small. The reduction in computational overhead is achieved by avoiding repeated O(qd) entropy-weight recalculations during stable operating periods.

### 4.3. Multi-Source Trust Fusion

In this section, i and j are redefined as the evaluating and target nodes, respectively.

#### 4.3.1. Direct Trust Evaluation

For target node j, the direct trust ${DT}_{ij}$ evaluated by node i is calculated from the current evidence values and their Fuzzy T-EWM weights:

(19) $${DT}_{ij}=\sum_{k=1}^{n}w_{k}\cdot E_{k}$$

where $E_{k}\in \left\{\eta ,T_{delay},\varepsilon ,p\left(t\right),R_{legit},S_{comp},S_{beh}\right\}$, and ($w_{k}$) denotes the weight assigned to the corresponding evidence metric.

The evidence comes from three layers. The H1 physical and communication layer includes the interaction success rate η, delay trust $T_{delay}$, energy-adaptation metric ε, and payload-consistency score p(t). These metrics reflect communication quality, resource status, and the consistency of the reported physical data.

The H2 network and routing-legitimacy layer uses $R_{legit}$ to check whether data are forwarded through authorized paths. The score decreases when a node attempts to transmit data to an address or port outside the local whitelist.

The H3 semantic and behavioral layer contains $S_{comp}$ and $S_{beh}$. The former checks whether the issued command belongs to the authorized operation set. The latter measures the deviation from the expected operating pattern.

#### 4.3.2. Robust Indirect Trust Aggregation Mechanism

In scenarios where node i has no prior interactions with node j (the cold-start problem), direct trust cannot be established. The system must rely on third-party recommendations. However, blindly averaging these paths or simply selecting the maximum values exposes the network to collusive good-mouthing, bad-mouthing, and Sybil attacks. To address these vulnerabilities, we introduce a robust credibility-weighted trimmed mean aggregation model.

Let $Path=\{{path}_{1},{path}_{2}\ldots ,{path}_{m}\}$ be the set of all possible recommendation paths from node i to node j. We first limit the valid path set ${Path}_{valid}$ to a strict 3-hop maximum to prevent routing loops and reduce calculation overhead:

(20) $${Path}_{valid}=\left\{{path}_{k}\in Path|\;hop({path}_{k})\leq 3\right\}$$

The trust value propagating down each valid path is the product of its adjacent direct trusts:

(21) $$trust({path}_{k})=\prod_{(x,y)\in {path}_{k}}{DT}_{xy}$$

Suppose that node i receives m recommendation scores from different trust paths. Directly selecting the highest recommendations can easily bias the initialization result. Therefore, the indirect trust ${RT}_{ij}$ is calculated in two steps.

First, each recommendation is weighted according to the credibility of the recommender node. Let $C_{r}\in \;[0,1]$ denote the current direct trust value of recommender r. The adjusted recommendation score is

(22) $$trust\prime \;({path}_{k})=C_{r}\times trust({path}_{k})$$

In this way, recommendations from highly trusted nodes contribute more to the final result.

Second, the set of m adjusted recommendation scores are sorted in ascending order ${trust\prime }_{\left(1\right)}\leq \;{trust\prime }_{\left(2\right)}\leq \cdots \leq {trust\prime }_{\left(m\right)}$. A trimming parameter τ is used to remove the highest and lowest scores (e.g., removing the top 10% and bottom 10% of the scores). The remaining recommendations are averaged to obtain the indirect trust value:

(23) $${RT}_{ij}=\frac{1}{m-2\tau }\sum_{k=\tau +1}^{m-\tau }{trust\prime }_{\left(k\right)}$$

This prevents a small number of extreme recommendations from dominating the initialization process.

#### 4.3.3. Comprehensive Trust Fusion

We dynamically fuse direct experience with indirect recommendations based on actual interaction history:

(24) $$T_{ij}=\alpha \cdot {DT}_{ij}+(1-\alpha )\cdot {RT}_{ij}$$

The weight α∈[0,1] scales linearly with the interaction frequency ς:

(25) $$\alpha =\{\begin{array}{c} \alpha_{min}\;\;,\;\varsigma =0\\ \alpha_{min}+(\alpha_{max}-\alpha_{min})\\ \alpha_{max}\;\;,\;\varsigma \geq \varsigma_{th} \end{array}\times \frac{\varsigma }{\varsigma_{th}}\;\;,\;0<\varsigma <\varsigma_{th}$$

When nodes have no interaction history, indirect recommendations receive the larger share of the fusion weight. As the number of direct interactions increases, the contribution of direct trust gradually becomes more prominent.

### 4.4. Asset-Driven Resilient Asymmetric Update

A symmetric update rule may allow a malicious node to recover its trust score too quickly after a short period of compliant behavior. This creates an opportunity for whitewashing and on–off attacks. ART uses an asymmetric update rule to reduce this risk. A significant trust reduction activates the Fast-Drop branch, whereas normal behavior follows a controlled recovery process.

#### 4.4.1. Asset Value Quantification

The operational importance of a device is represented by the normalized asset value $V_{s}$. It is calculated from the confidentiality, integrity, and availability attributes of node s:

(26) $$V_{s}=\rho_{A}A_{s}+\rho_{I}I_{s}+\rho_{C}C_{s}$$

The confidentiality $C_{s}$, integrity $I_{s}$, and availability $A_{s}$ indicators are normalized to the range [0, 1]. Their weights are selected according to the role of the device in the industrial process and satisfy $\rho_{A}+\rho_{I}+\rho_{C}=1$. For example, integrity is usually assigned greater importance for a PLC involved in process control.

The simulations use fixed asset values to maintain controlled experimental conditions. These values are not treated as permanent settings for practical deployment. The underlying $A_{s}$, $I_{s}$, and $C_{s}$ attributes can be recalibrated periodically according to the asset inventory, device role, process dependency, and operational risk profile. The risk-assessment procedure and target security levels specified in IEC 62443-3-2 [35] may be used as reference inputs during this process.

#### 4.4.2. Resilient Asymmetric Update Model

The dynamic trust score ${T(t)}_{new}$ is updated according to the current aggregated score and the trust value from the previous evaluation slot:

(27) $${T(t)}_{new}=\{\begin{array}{c} \left(1-\theta \left(s\right)\right)\cdot T\left(t-1\right)+\theta \left(s\right)\cdot T\left(t\right),if\;T\left(t\right)\geq T\left(t-1\right)-\Delta_{tol}\\ \lambda \cdot T\left(t\right)+\left(1-\lambda \right)\cdot T\left(t-1\right),if\;T\left(t\right)<T\left(t-1\right)-\Delta_{tol} \end{array}$$

Here T(t) is the real-time aggregated score and T(t−1) is the historical trust score.

The tolerance margin $\Delta_{tol}$ is introduced to limit unnecessary penalties caused by routine fluctuations. When the reduction in the aggregated score remains within this margin, the node follows the recovery branch. Minor variations caused by delay jitter, packet loss, or short-term measurement noise therefore do not immediately activate the Fast-Drop branch.

A larger reduction is treated differently. When the score decrease exceeds $\Delta_{tol}$, the penalty factor λ assigns greater weight to the current abnormal score. The trust value then decreases more rapidly, allowing the isolation mechanism to respond to a substantial deviation.

A strict penalty mechanism can improve security, but it may also delay the recovery of critical devices after temporary disturbances. In industrial environments, communication jitter, packet loss, and short-term network fluctuations do not necessarily indicate malicious behavior. To distinguish these situations, trust recovery is adjusted through the context-aware coefficient θ(s):

(28) $$\theta (s)=max\left(\theta_{min},\theta_{0}\cdot \left(1-\kappa \cdot V_{s}\right)\right)$$

where $\theta_{0}$ is the baseline recovery coefficient, κ∈[0,1] denotes the anomaly-severity factor, and $\theta_{min}$ is the minimum recovery bound.

The severity factor κ depends on the source and persistence of the observed deviation. Short-term H1 fluctuations, such as temporary delay variation or packet loss, are assigned a relatively small κ. In this case, the recovery coefficient remains close to $\theta_{0}$, and the node can return to its normal trust range without an extended delay.

A higher value of κ is used when the trust reduction is associated with persistent routing violations or H3 semantic and behavioral evidence, such as unauthorized commands or abnormal operating patterns. The asset value $V_{s}$ then has a stronger influence on the recovery coefficient. As a result, a high-value device recovers more cautiously after a potentially malicious event.

The lower bound $\theta_{min}$ prevents the recovery coefficient from approaching zero. Gradual recovery therefore remains possible after a severe penalty, provided that the node continues to exhibit normal behavior.

The value of κ depends on the nature of the observed deviation. It remains low for temporary communication disturbances and approaches 1 for serious semantic or behavioral violations.

Scenario B uses the severe-anomaly case κ=1 to evaluate asset-driven Slow-Rise recovery after malicious behavior.

Algorithm 2 is the pseudocode of Asset-Driven Resilient Asymmetric Trust Update.
**Algorithm 2.** Asset-Driven Resilient Asymmetric Trust Update     **Input:** Current aggregated score T(t), Historical trust T(t−1), Device attributes $\left\{A_{s},I_{s},C_{s}\right\}$, Base weight $\theta_{0}$, Penalty factor λ, Anomaly-severity factor κ, Tolerance margin $\Delta_{tol}$, Minimum recovery bound $\theta_{min}$.
  **Output:** Updated Dynamic Device Trust Score ${T(t)}_{new}$.
**1. Quantify** asset value $V_{s}=\rho_{A}A_{s}+\rho_{I}I_{s}+\rho_{C}C_{s}$ using Equation (26);
**2. Calculate** dynamic recovery coefficient θ(s) using Equation (28);
**3. If**
$T\left(t\right)\geq T\left(t-1\right)-\Delta_{tol}$
**then**      // Trust recovery phase 
**4.  Update**
${T(t)}_{new}=\left(1-\theta \left(s\right)\right)\cdot T\left(t-1\right)+\theta \left(s\right)\cdot T\left(t\right)$ using Equation (27);
**5. Else**     // Anomaly detected: Trust penalty phase 
**6.  Update**
${T(t)}_{new}=\lambda \cdot T\left(t\right)+\left(1-\lambda \right)\cdot T\left(t-1\right)$ using Equation (27)
**7. End If**
**8. Store**
${T(t)}_{new}$ as the reference for the next cycle; 
**9. Return**
${T(t)}_{new}$


### 4.5. Asset-Aware Cross-Plane Regulation

The asset value $V_{s}$ is not used as an independent trust score. It acts as a control variable that adjusts the response of the horizontal evidence plane. In ART, this adjustment is applied to the trigger threshold and the recovery process.

The trigger threshold in Equation (18) changes with $V_{s}$. A high-value node is assigned a smaller threshold, so a relatively small evidence variation can activate weight recalculation. This setting is useful for devices such as PLCs, where a minor abnormal change may have a larger operational impact. For auxiliary nodes, a larger tolerance range avoids frequent recalculation under routine fluctuations.

The asset value also affects trust recovery. According to Equation (28), the recovery coefficient depends on both $V_{s}$ and the anomaly-severity factor κ. When the trust reduction is mainly caused by temporary delay, packet loss, or other short-term communication disturbances, κ remains low and the node can return to its normal state more quickly. A different response is applied when semantic or behavioral evidence indicates a more serious violation. In this case, κ increases, and the recovery rate of a high-value node is reduced.

This design separates two issues that should not be treated in the same way: the importance of the device and the severity of its current behavior. The horizontal plane continues to evaluate real-time evidence, while the vertical plane adjusts how sensitive the framework is to evidence changes and how cautiously trust is restored after a penalty.

## 5. Simulation and Performance Evaluation

### 5.1. Simulation Setup and Baselines

#### 5.1.1. Simulation Parameters and Network Topology

Our simulated IIoT edge network tracks N=50 different nodes, which interact continuously in T=1000 time slots. Industrial devices differ in their operational roles and security criticality. The simulated network therefore includes four asset categories. Using Equation (26), we map their physical importance into a specific $V_{s}$ value:

Control Nodes (such as PLC controllers): 10 nodes. As core assets, set $V_{s}\approx 0.95$.

Production Nodes (such as computer numerical control (CNC) machines): 15 nodes, as high-value assets, set $V_{s}\approx 0.85$.

Monitoring Nodes (such as environmental sensors): 15 nodes with a medium asset value of $V_{s}\approx 0.60$.

Auxiliary Nodes (such as smart lighting): 10 nodes are given a very low asset value of $V_{s}\approx 0.30$.

To construct a realistic industrial simulation environment, the baseline parameters were configured according to the statistical characteristics reported in publicly available IIoT and Hardware-in-the-Loop (HIL) datasets, including the HAI (HIL-based Augmented ICS Security Dataset) [36] and the SWaT (Secure Water Treatment) dataset [37]. The hierarchical asset categories (Control, Production, Monitoring, and Auxiliary) and the sensor variance settings (e.g., $\sigma_{v}=0.02$) were derived from these industrial references. To evaluate the behavior of the framework under adversarial conditions, synthetic attack traces, including stealthy FDI injections and whitewashing attacks, were introduced into the baseline dataset.

The Fast-Drop penalty factor was set to λ=0.85 to provide a strong response to severe semantic violations. The baseline recovery coefficient $\theta_{0}$ was set to 0.5. The quantitative sensitivity analysis of $\theta_{0}$ is detailed in Section 5.2.6.

The main algorithmic parameters were determined through sensitivity analysis. When $\theta_{0}$ exceeded 0.6, malicious nodes could regain trust too quickly after a penalty. When $\theta_{0}$ fell below 0.3, trust recovery became noticeably slower even after temporary non-malicious disturbances. Therefore, $\theta_{0}=0.5$ was selected as a compromise between security and availability. For indirect trust aggregation, the interaction threshold was configured as $\varsigma_{th}=10$. For the Fuzzy T-EWM, the anomaly trigger threshold was set to $\Gamma_{base}=0.15$. This value provides a balance between detection responsiveness and computational overhead. The asset value $V_{s}$ was calculated according to the CIA-based quantification model in Equation (26).

#### 5.1.2. Baseline Models

ART is compared with three baseline models to evaluate semantic-attack detection, whitewashing resistance, robustness against collusive recommendations, and computational overhead:

- Baseline 1 (Classic Beta Model): This is a traditional subjective model, which is calculated entirely by the Beta distribution of the basic communication success rate. It uses a rigid and symmetrical update method, without any asset awareness.
- Baseline 2 (Standard EWM Model): This recalculates the entropy-weight matrix at each evaluation slot and does not reuse cached weights during stable periods.
- Baseline 3 (Conventional Path-Averaging Trust Model): It simply averages all the paths that can be found to aggregate third-party recommendations. It lacks a rigorous robust filtering mechanism, leaving it vulnerable to orchestrated collusive behaviors such as good-mouthing and bad-mouthing attacks.
- Additional Comparative Strategy (Top-k Average Recommendation Strategy): Scenario C also includes a Top-k average strategy for comparison. Let $P_{ij}$ denote the set of valid recommendation paths. The path trust values are sorted in descending order, and the first $k=min\left(3,\left|P_{ij}\right|\right)$ values are averaged:

(29) $${RT}_{ij}^{Top-k}=\frac{1}{k}\sum_{l=1}^{k}{trust}_{\left(l\right)}$$

If no valid recommendation path is available ($\left|P_{ij}\right|=0$), indirect trust initialization is deferred until at least one valid path is observed. This strategy gives priority to high-valued recommendations but does not filter abnormal scores. It may therefore be affected by collusive recommendation manipulation.

- Proposed (ART Framework): This is our full deployment architecture, featuring cross-layer physical-semantic checking (H1 to H3), Fuzzy T-EWM for triggered lightweight aggregation, robust credibility-weighted trimmed mean filtering for indirect trust, and resilient asymmetric updates driven by $V_{s}$.

### 5.2. Simulation Results and Discussion

To evaluate the contribution of each component in ART, the simulations were organized into several dedicated scenarios. Instead of only reporting the overall performance, each core module was examined separately.

Scenario A evaluates whether the H1 payload-consistency mechanism can detect stealthy FDI attacks that do not produce obvious communication-level anomalies. Scenario B evaluates the asset-aware mechanism together with the asymmetric penalty and recovery strategy under whitewashing and on–off attacks. Scenario C examines the credibility-weighted indirect trust mechanism under collusive recommendation attacks, including Sybil-style and good-mouthing behaviors. Scenario D evaluates the computational overhead of the complete framework.

For comparison, ART was evaluated against several representative trust-management approaches, including the Classic Beta model, the Standard EWM method, conventional path-averaging, and the Top-k Average Recommendation strategy. The parameters of these baseline models were configured according to their commonly adopted settings in existing IIoT trust-management studies.

#### 5.2.1. Scenario A: Defense Against Stealthy False Data Injection (FDI) Attacks

This scenario considers a stealthy False Data Injection (FDI) attack, which has also been examined in recent sensor-network security studies [38]. A compromised node begins to report altered sensor values at t=200. The packet length remains unchanged, with $l=l_{th}$, and the communication success rate stays close to its normal level. The injected sensor value is increased by 8% relative to the reference baseline $\hat{v}$.

**Figure 2:**
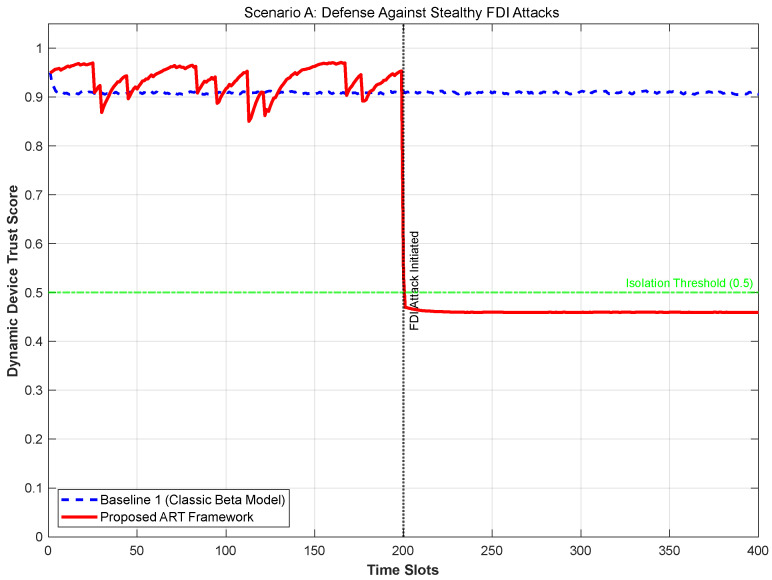
Trust evolution of edge nodes under stealthy False Data Injection (FDI) attack.

During the initial stable phase, both ART and Baseline 1 maintain high trust scores, as shown in Figure 2. After the attack begins, the two curves show different responses.

Baseline 1 continues to report a high trust score because it relies mainly on the interaction success rate η. Since the communication pattern changes very little in this experiment, the modified sensor value is not reflected in the baseline result.

ART also evaluates payload consistency. In Equation (6), the packet-length term remains zero, but the difference between v and $\hat{v}$ increases after the FDI attack begins. The deviation is normalized by $\sigma_{v}=0.05$, and the physical-residual weight is set to $\omega_{v}=0.8$. The payload-consistency score p(t) therefore decreases.

The reduction in the aggregated score activates the Fast-Drop branch in Equation (27). With λ=0.85, the ART trust score falls below the isolation threshold shortly after t=200.

These results show that communication indicators alone may not reveal payload-level manipulation. The physical residual allows ART to respond when the reported sensor value deviates from its reference baseline.

#### 5.2.2. Scenario B: Mitigation of Whitewashing (On–Off) Attacks via Asset-Driven Asymmetric Updates

Whitewashing and on–off attacks exploit the recovery process of a trust model. A malicious node may alternate between abnormal and compliant behavior, using the compliant intervals to restore its trust score before launching another attack. Scenario B examines whether the asymmetric update rule can limit this behavior.

Two nodes with different operational roles are considered: a high-value control node with $V_{s}=0.95$ and an auxiliary node with $V_{s}=0.30$. The simulation is divided into three periods. Both nodes behave normally before t=100. Malicious interactions are introduced during 100≤t<200. From t=200, the nodes return to compliant behavior, representing the whitewashing phase.

Scenario B represents a severe malicious-behavior case. The anomaly-severity factor is therefore set to κ=1. Under this condition, Equation (28) becomes an asset-driven Slow-Rise setting $\theta \left(s\right)=\max\left\{\theta_{min},\theta_{0}\left[1-V_{s}\right]\right\}$. This configuration is used to compare the recovery behavior of nodes with different asset values after the malicious phase.

**Figure 3:**
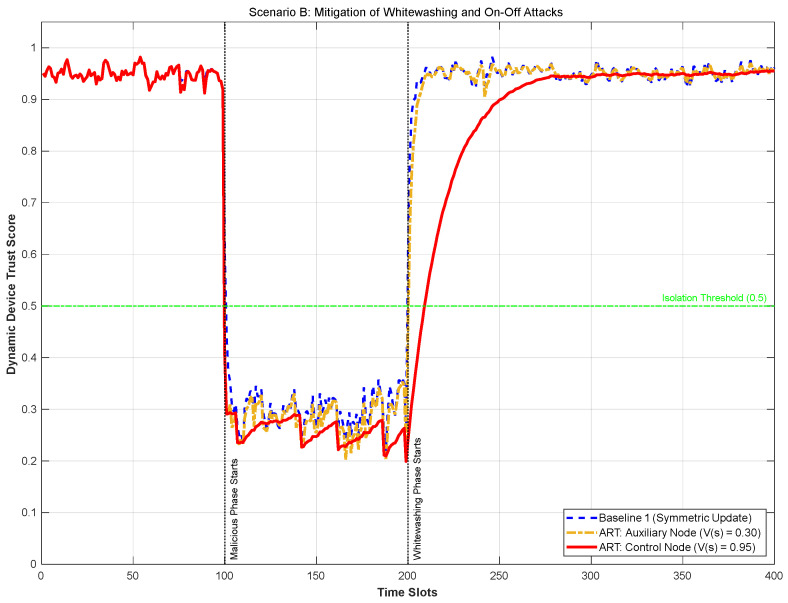
Representative trust trajectories under a whitewashing and on–off attack scenario. The high-value control node follows a slower trust-recovery process than the auxiliary node and the symmetric-update baseline.

As shown in Figure 3, the trust scores decrease rapidly after the malicious phase begins. The Fast-Drop branch is activated because the reduction in the aggregated score exceeds the tolerance margin. With the penalty factor set to λ=0.85, the trust values fall below the isolation threshold shortly after the abnormal behavior is observed.

The difference between the update rules becomes clearer after t=200. Baseline 1 restores the trust score quickly once compliant behavior resumes. The auxiliary node evaluated by ART also recovers relatively quickly because its asset value is lower. The high-value control node follows a slower trajectory. Its trust score rises gradually and remains below the other two curves for a longer period.

This behavior is governed by the recovery coefficient in Equation (28). Under this severe-anomaly setting, the asset value $V_{s}\;$ has a stronger effect on the recovery coefficient of the control node. Its recovery coefficient becomes smaller, and a longer sequence of compliant behavior is required before the trust score returns to the normal range. The node is not permanently excluded; recovery remains possible after sustained normal behavior.

The slow recovery shown in Figure 3 should not be interpreted as the default response to every short-term disturbance. Temporary packet loss, delay variation, or routine network jitter mainly affect H1 communication evidence. In these cases, κ remains low, and the recovery coefficient stays closer to its baseline value. The node can therefore recover more quickly after a non-malicious fluctuation.

The lower bound $\theta_{min}$ in Equation (28) prevents the recovery coefficient from approaching zero under severe penalty conditions. This preserves gradual recovery while avoiding an excessively prolonged recovery period for critical devices.

The results show that ART distinguishes the severity of the observed behavior from the operational importance of the affected device. This limits rapid trust restoration after a serious violation without imposing the same recovery delay on routine communication disturbances.

#### 5.2.3. Scenario C: Defense Against Collusive Good-Mouthing and Sybil-Style Attacks in Cold-Start Scenarios

When a new node enters the network (ς=0), no direct interaction history is available. The trust evaluation therefore depends on recommendations provided by other nodes. To evaluate the robustness of the indirect trust mechanism, we simulated a collusive good-mouthing attack during the cold-start stage. In this scenario, the target node has an actual trust value of 0.25, while 40% of the recommenders provide artificially inflated trust scores of 0.95.

**Figure 4:**
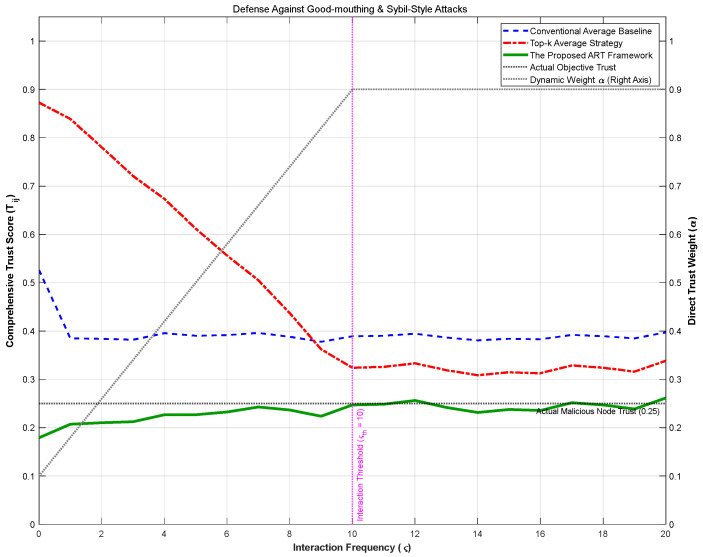
Defense against collusive good-mouthing and sybil-style attacks in cold-start scenarios via credibility-weighted trimmed mean filtering and dynamic weighting.

Figure 4 compares the behavior of different aggregation strategies under this setting.

As shown in Figure 4, the Top-k Average Recommendation strategy assigns a high initial trust value to the malicious node because it relies heavily on the largest recommendation scores. The Average method performs slightly better, but the final result is still affected by the manipulated recommendations. In both cases, the initial trust estimate deviates noticeably from the actual trust level.

The ART framework combines recommender credibility weighting with trimmed mean filtering. Recommendations from low-trust nodes contribute less to the aggregation process, while extreme scores are removed before the final calculation. Consequently, the estimated trust value remains close to the actual trust baseline throughout the cold-start stage.

Another observation can be obtained from the dynamic weight parameter α shown in Figure 4. During the early interactions, indirect recommendations contribute to trust initialization. As the interaction count increases, the influence of direct observations gradually becomes stronger. Once ς reaches the predefined threshold $\varsigma_{th}=10$, the trust evaluation is dominated by direct evidence rather than third-party recommendations.

These results indicate that indirect trust initialization is particularly vulnerable to manipulated recommendations when simple averaging or maximum-value selection is used. By combining credibility weighting, trimmed mean filtering, and gradual transition toward direct observations, ART reduces the impact of collusive recommendation attacks during the cold-start phase.

#### 5.2.4. Scenario D: Edge Lightweight Overhead Analysis

Industrial edge nodes lack the energy resources for repeated entropy-weight recalculation. This final scenario compares the accumulated computational cost of Fuzzy T-EWM relative to Standard EWM (Baseline 2). We ran the simulation over 1000 time slots and introduced network volatility to trigger our anomaly threshold ($\Gamma_{base}=0.15$).

**Figure 5:**
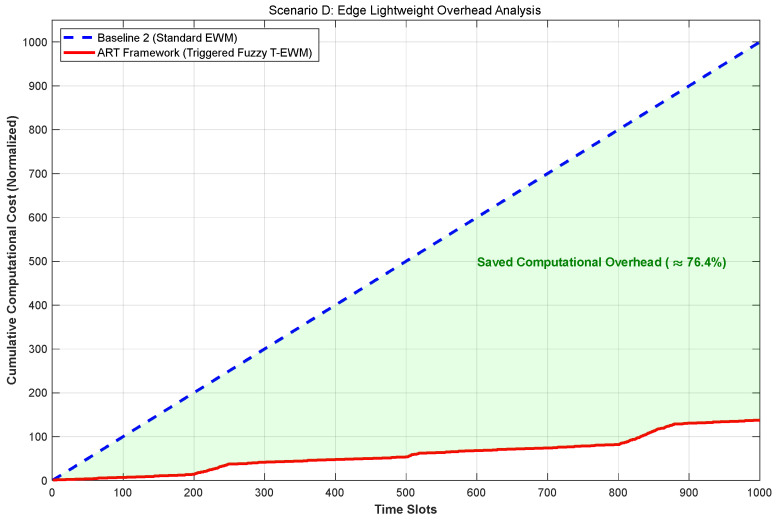
Accumulated computational cost of the Standard EWM baseline and the triggered Fuzzy T-EWM mechanism over 1000 evaluation slots.

Figure 5 shows different growth patterns for the two methods.

Baseline 2 exhibits a steep and continuous linear climb. Because Standard EWM lacks state-awareness, the Standard EWM baseline recalculates the entropy weights at every evaluation slot, so its cumulative cost increases continuously. This slot-by-slot recalculation strategy increases the processing burden of edge nodes.

In contrast, our framework produces a flat, step-like growth pattern. Whenever the network is stable and real-time evidence fluctuation stays below the threshold (ΔΕ<Γ), ART bypasses the matrix recalculation. Standard EWM recalculates entropy weights at each evaluation slot, so its cumulative cost increases continuously. Fuzzy T-EWM reuses cached weights during stable periods and recalculates the weights only when the trigger condition is satisfied. This produces the step-like trajectory in Figure 5. By pruning redundant operations, our Triggered Calculation Mechanism reduces the cumulative computational overhead by 76.4% compared to the Standard EWM.

From the computational perspective, the Standard EWM method (Baseline 2) performs a complete O(qd) weight update at every time slot. In our overhead analysis, this operation is treated as the reference cost and normalized to 1.0. In contrast, ART reuses the previously calculated weights when the evidence fluctuation remains below the trigger threshold. During stable operating periods, ART reuses the cached weights instead of recalculating the full entropy-weight matrix. Although retrieving the cached weights requires only O(1) time, the complete stable-slot evaluation remains O(d) because the current evidence vector must still be compared with the cached reference state and aggregated using the retained weights. By contrast, a triggered recalculation requires O(qd) time.

To provide a runtime-oriented illustration, a complete entropy-weight recalculation is assigned a reference cost of 8.9 ms per slot. This value is used only to map the normalized computational overhead to an illustrative processing-time estimate; it is not a direct measurement from a physical testbed. Based on the 76.4% relative reduction observed in the simulation, the estimated amortized processing time of Fuzzy T-EWM is 8.9×(1−0.764)≈2.1 ms per slot.

These values are runtime estimates rather than direct measurements from a physical testbed. The actual processing time depends on the hardware platform, software implementation, matrix dimensions, and runtime environment. Device-level profiling is therefore required before making a real-time deployment claim.

#### 5.2.5. Scenario E: H1-Focused Evaluation Under a Near-Complete Black-Hole Attack

To evaluate the response of the H1 communication-evidence layer under persistent packet dropping, we introduced a near-complete black-hole attack scenario. The malicious node operates normally before time slot 150 and subsequently drops nearly all forwarded packets. The experiment was repeated over 100 Monte Carlo runs.

**Figure 6:**
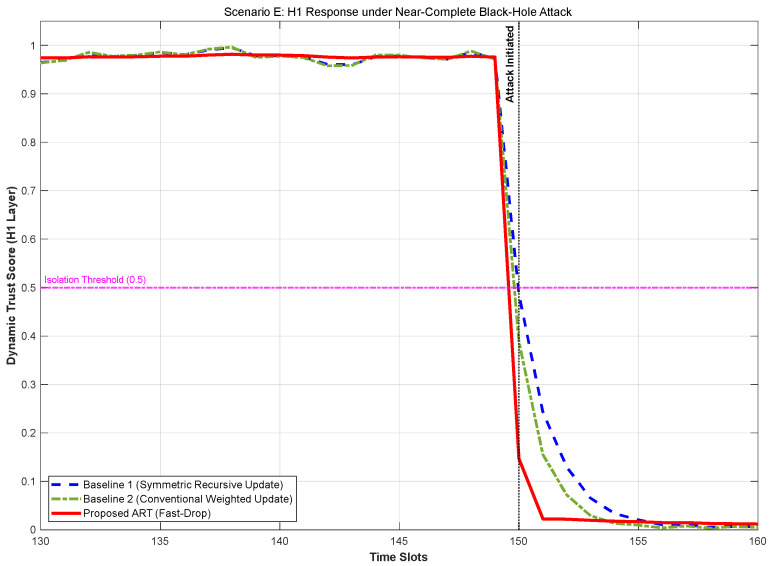
Representative trust trajectories in Scenario E under a near-complete black-hole packet-dropping attack.

Figure 6 presents a representative trust trajectory. After the attack begins, all evaluated methods reduce the trust score below the isolation threshold. ART produces the steepest immediate decline because the Fast-Drop rule assigns a larger weight to the abnormal communication evidence. As summarized in Table 2, ART achieves a detection rate of 100.0%, a run-level false isolation rate of 0.0% before attack onset, and a mean isolation delay of 1.00 evaluation slot. These results indicate that the H1 evidence plane responds rapidly to near-complete packet-dropping behavior without introducing false isolation during normal operation.

The tolerance margin $\Delta_{tol}$ limits the influence of normal network jitter on the Fast-Drop mechanism. In the evaluated configuration, $\Delta_{tol}=0.05$, while the standard deviation of the normal PDR fluctuation is 0.02. Under a steady-state approximation, the corresponding per-slot false-activation probability is Φ(−2.5)≈0.0062=0.62%. In the Monte Carlo evaluation, the measured event-level false Fast-Drop activation rate is 0.514%. The mean temporary trust reduction after a false activation is 0.0476, and the maximum observed reduction is 0.0603. Although 54.0% of the runs contain at least one short-lived false activation, the run-level false isolation rate before attack onset remains 0.0%. This distinction indicates that occasional jitter-induced activations do not cause healthy nodes to fall below the isolation threshold.

#### 5.2.6. Quantitative Performance Summary

To compare the different trust-management strategies, several quantitative metrics were extracted from the simulation results. Table 3 summarizes the performance of all evaluated frameworks. Runtime-oriented estimates are reported only for Standard EWM and ART, as these two methods share the same entropy-weight recalculation reference.

The results show that ART achieves the highest detection rate against semantic attacks while maintaining the lowest false alarm rate among the compared approaches. In Scenario A, the proposed framework reaches a detection rate of 98.7%, whereas the Classic Beta model is unable to identify semantic-layer manipulations. In Scenario C, the Path-Averaging method is more susceptible to collusive recommendations, resulting in a higher false alarm rate. Under the evaluated stable operating conditions, ART limits the false alarm rate to 1.89%.

For whitewashing and on–off attacks in Scenario B, the asset-aware asymmetric update rule reduces the isolation delay to fewer than two evaluation slots. The triggered weighting mechanism also avoids repeated entropy-weight recalculation during stable periods. Based on the runtime-oriented mapping described above, the estimated amortized processing time of ART is approximately 2.1 ms per slot. This value is an estimate rather than a direct measurement from a physical testbed. The results indicate that ART improves the response to malicious behavior while limiting repeated processing at the edge.

#### 5.2.7. Parameter Sensitivity Analysis

**Figure 7:**
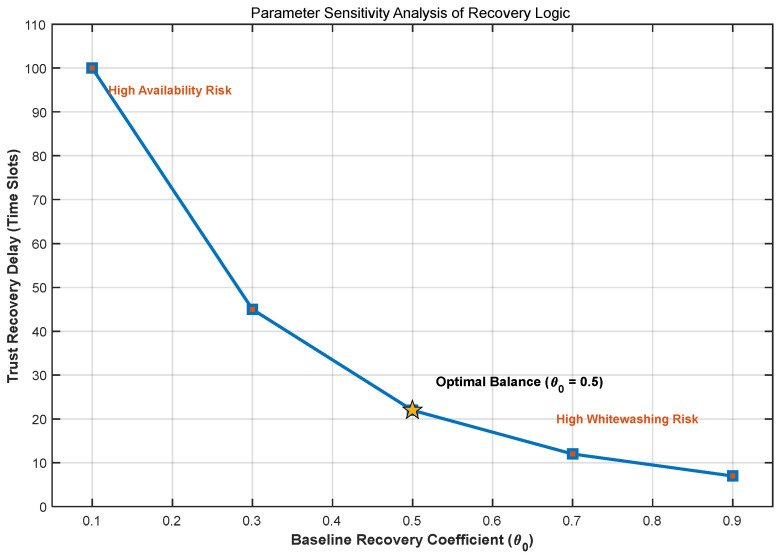
Parameter sensitivity analysis of the baseline recovery coefficient ($\theta_{0}$) based on empirical trust recovery delay. The pentagram marker highlights the selected optimal baseline configuration ($\theta_{0}=0.5$) that achieves a trade-off between prolonged recovery delays (high availability risk) and overly rapid trust restoration (high whitewashing risk).

The recovery coefficient $\theta_{0}$ affects the trust recovery speed after a penalty. Figure 7 shows the recovery delay under different $\theta_{0}$ settings.

As $\theta_{0}$ increases from 0.1 to 0.9, the recovery delay decreases from approximately 100 slots to 7 slots. Specifically, the delay is about 45 slots at $\theta_{0}=0.3$, 22 slots at $\theta_{0}=0.5$, and 12 slots at $\theta_{0}=0.7$.

Very small $\theta_{0}$ values prolong the recovery process and may reduce availability after temporary disturbances. In contrast, large $\theta_{0}$ values shorten the observation period of penalized nodes and weaken the suppression effect on whitewashing behavior.

Considering both factors, $\theta_{0}=0.5$ was selected for the remaining experiments. Under this setting, the recovery delay remains moderate while preserving resistance against rapid trust restoration.

The baseline trigger threshold $\Gamma_{base}$ determines the sensitivity of the entropy-weight recalculation mechanism. It is used to derive the node-specific threshold Γ(s) through Equation (18). To examine the influence of this parameter, we varied $\Gamma_{base}$ from 0.05 to 0.25 under the same workload and attack conditions.

**Figure 8:**
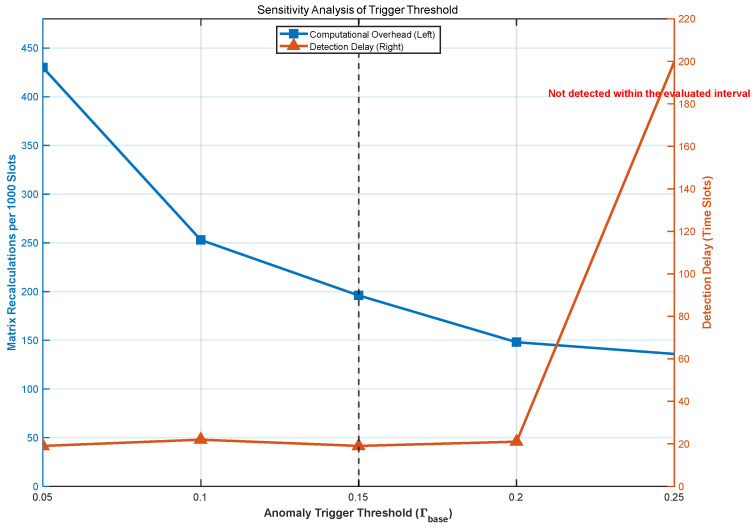
Parameter sensitivity analysis of the anomaly trigger threshold ($\Gamma_{base}$) based on computational overhead and detection delay.

Figure 8 shows the relationship between the baseline threshold, matrix recalculation frequency, and detection delay. A smaller threshold makes the framework more responsive to minor evidence changes. When $\Gamma_{base}=0.05$, the entropy weights are recalculated 430 times per 1000 slots. The recalculation count decreases to 253 at $\Gamma_{base}=0.10$, 196 at $\Gamma_{base}=0.15$, and 148 at $\Gamma_{base}=0.20$. Over this range, the detection delay remains between 19 and 22 slots. When the threshold is further increased to 0.25, the gradual evidence changes caused by the stealthy drift attack are not sufficient to activate the recalculation mechanism within the evaluated interval.

**Figure 9:**
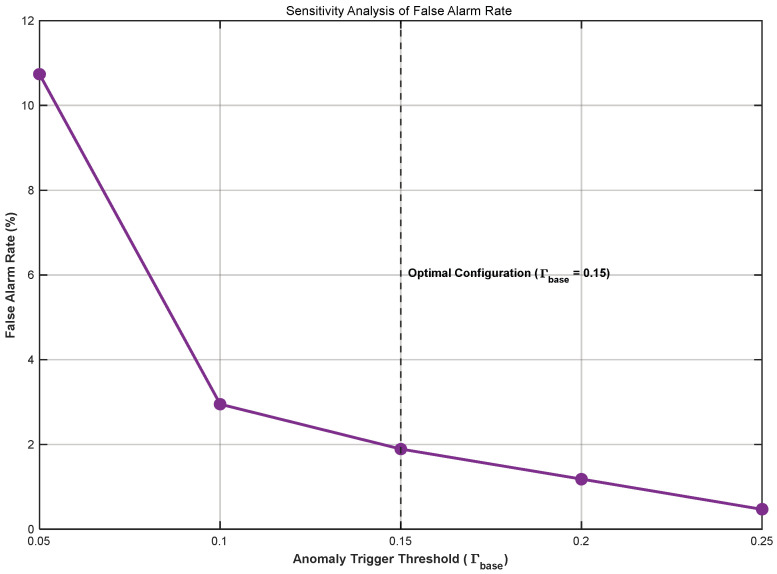
Parameter sensitivity analysis of the anomaly trigger threshold ($\Gamma_{base}$) based on false alarm rate.

Figure 9 presents the corresponding false alarm rate under stable operating conditions. With $\Gamma_{base}=0.05$, routine fluctuations lead to a false alarm rate of 10.74%. The rate decreases to 2.95% at $\Gamma_{base}=0.10$ and 1.89% at $\Gamma_{base}=0.15$. Further increases reduce the false alarm rate to 1.18% and 0.47% at thresholds of 0.20 and 0.25, respectively. However, the lower false alarm rate at larger thresholds is accompanied by a reduced response margin for gradual deviations.

Considering the recalculation frequency, detection delay, and false alarm rate together, $\Gamma_{base}=0.15$ is used as a balanced configuration in the subsequent experiments. This setting avoids excessive recalculations under routine fluctuations while retaining sensitivity to slow-changing attacks.

The Fast-Drop penalty factor λ controls the reduction in trust after a severe semantic violation. A smaller value leads to a slower decline, whereas a larger value increases the penalty rate. In the subsequent experiments, λ=0.85 is used to provide a rapid trust reduction after a severe violation is detected.

## 6. Conclusions

This study proposes an ART framework for IIoT edge networks. Its main contribution is to extend conventional behavior-centered trust evaluation into an asset-aware and context-dependent trust-regulation model. ART separates cyber-behavioral credibility from physical asset criticality and links these two dimensions through the trust-update process. As a result, nodes with different operational roles are not treated uniformly when anomalous behavior occurs. Asset criticality is incorporated directly into the trust dynamics rather than used only as an external risk label.

The framework combines this dual-plane structure with cross-layer H1–H3 evidence fusion. Physical and semantic consistency checks are included to identify deviations that may not be captured by communication indicators alone. To reduce the processing burden at the edge, Fuzzy T-EWM recalculates entropy weights only when the evidence fluctuation exceeds the asset-aware trigger threshold. During stable periods, the cached weights are reused. ART also introduces a context-aware asymmetric update rule. Significant deviations activate the Fast-Drop branch, while the recovery process is controlled by the asset value, anomaly severity, and tolerance margin $\Delta_{tol}$. This formulation provides a structured way to balance threat suppression and service availability.

The simulation results show that ART detects stealthy FDI attacks, limits whitewashing recovery, and reduces the accumulated computational overhead by 76.4% compared with the Standard EWM baseline. The credibility-weighted trimmed mean mechanism also limits the influence of collusive recommendation manipulation during the cold-start phase. In addition, the H1-focused black-hole evaluation shows that ART responds rapidly to near-complete packet-dropping behavior. Across 100 Monte Carlo runs, the malicious node is isolated within the first evaluation slot, while the run-level false-isolation rate before attack onset remains 0.0%. The jitter analysis further shows that occasional short-lived Fast-Drop activations do not cause healthy nodes to fall below the isolation threshold.

Future work will focus on three directions. First, dynamic asset recalibration will be investigated for industrial environments in which device roles and operational dependencies change over time. Second, the framework will be extended to protocol-level and multi-stage attack scenarios, including more complex routing and identity-management behaviors. Third, we plan to integrate distributed ledger technology for tamper-resistant trust-evidence management and evaluate ART on a Hardware-in-the-Loop smart-manufacturing testbed.

## Figures and Tables

**Table 1:** Qualitative comparison of representative trust-evaluation approaches.

Feature	Rule-Based Fuzzy Trust/Security Models [18,19]	Standard EWM [28]	ML-Based Models [26,30,31,32]	Proposed ART Framework
Weight Allocation	Often based on predefined rules or expert-defined parameters	Data-driven entropy weighting	Learned from training data	Entropy-based data-driven weighting
Weight Update Mode	Model-dependent	Recalculated at each evaluation slot in the baseline implementation	Model-dependent; may require repeated inference	Event-triggered update with cached weights
Edge-Processing Burden	Model-dependent	Increased burden under repeated recalculation	May increase due to inference cost	Reduced burden during stable periods
Asset Awareness	Usually not explicitly modeled	Not included	Depends on selected features	CIA-based asset value ($V_{s}$)
Trust-Update Logic	Model-dependent	Not explicitly modeled	Model-dependent	Context-aware asymmetric update
Detection Scope	Depends on selected indicators	Depends on input indicators	Depends on training features	Cross-layer H1–H3 evidence fusion
Noise Tolerance	Depends on parameter settings	Depends on input preprocessing	Depends on model design and training data	Adaptive threshold and tolerance margin $\Delta_{tol}$

Note: The table compares representative implementations of the listed approaches. In the Standard EWM baseline, entropy weights are recalculated at each evaluation slot. ART reuses cached weights during stable periods and updates them only when the trigger condition is met.

**Table 2:** Quantitative Results of the H1-focused Evaluation under a near-complete black-hole attack.

Method	Detection Rate	Run-Level False Isolation Rate Before Attack	Mean Isolation Delay
Symmetric Recursive Update	100%	0.0%	1.02 slots (SD = 0.14)
Conventional Weighted Update	100%	0.0%	1.00 slot (SD = 0.00)
Proposed ART (Fast-Drop)	100%	0.0%	1.00 slot (SD = 0.00)

Note: The experiment was repeated over 100 Monte Carlo runs. An isolation delay of 1.00 slot indicates that the trust score falls below the isolation threshold within the first evaluation slot after attack onset. For ART, the event-level false Fast-Drop activation rate under normal jitter is 0.514%. These short-lived activations do not result in false isolation.

**Table 3:** Quantitative Performance Metrics of Evaluated Frameworks.

Framework	Detection Rate (FDI)	False-Alarm Rate	Isolation Delay After Attack Onset	Estimated Amortized Processing Time/Slot
Baseline 1 (Classic Beta)	0.0% (Blind)	4.8%	>30 slots	N/A
Baseline 2 (Standard EWM)	89.2%	6.5%	15 slots	8.9 ms
Baseline 3 (Path-Averaging)	42.5%	8.2%	N/A	N/A
Proposed ART Framework	98.7%	1.89%	<2 slots	2.1 ms

Note: The processing-time values for Standard EWM and ART are illustrative runtime-oriented estimates derived from the normalized overhead analysis. They are not direct measurements from a physical testbed. Equivalent runtime mappings were not established for the remaining baselines; therefore, the corresponding entries are reported as N/A.

## Data Availability

The original contributions presented in this study are included in the article. Further inquiries can be directed to the corresponding author.

## Abbreviations

The following abbreviations are used in this manuscript:

APC	Article Processing Charge
ART	Asset-Aware Resilient Trust
CIA	Confidentiality, Integrity, and Availability
CNC	Computer Numerical Control
EWM	Entropy Weight Method
FDI	False Data Injection
Fuzzy T-EWM	Fuzzy Triggered-Entropy Weight Method
HAI	HIL-based Augmented ICS Security Dataset
HIL	Hardware-in-the-Loop
ICS	Industrial Control System
IIoT	Industrial Internet of Things
IP	Internet Protocol
IT	Information Technology
ML	Machine Learning
MTD	Moving Target Defense
OT	Operational Technology
PDR	Packet Delivery Ratio
PLC	Programmable Logic Controller
SWaT	Secure Water Treatment
